# Emotions and courtship help bonded pairs cooperate, but emotional agents are vulnerable to deceit

**DOI:** 10.1073/pnas.2308911120

**Published:** 2023-11-10

**Authors:** Suzanne Sadedin, Edgar A. Duéñez-Guzmán, Joel Z. Leibo

**Affiliations:** ^a^Independent Researcher, Abbots Langley WD5 0QS, United Kingdom; ^b^Google DeepMind, London EC4A 3TW, United Kingdom

**Keywords:** pair bond, cooperation, courtship, emotion, deceit

## Abstract

Coordinated pair bonds are common in birds and also occur in many other taxa. How do animals solve the social dilemmas they face in coordinating with a partner? We developed an evolutionary model to explore this question, based on observations that a) neuroendocrine feedback provides emotional bookkeeping which is thought to play a key role in vertebrate social bonds and b) these bonds are developed and maintained via courtship interactions that include low-stakes social dilemmas. Using agent-based simulation, we found that emotional bookkeeping and courtship sustained cooperation in the iterated prisoner’s dilemma in noisy environments, especially when combined. However, when deceitful defection was possible at low cost, courtship often increased cooperation, whereas emotional bookkeeping decreased it.

## Significance

Well-coordinated pair bond relationships occur in many species, humans included. Decisions in these relationships may be influenced by persistent emotions toward a partner (emotional bookkeeping). Here, we use a simulation model to show that animals guided by emotional bookkeeping can evolve cooperative pair bonds that are resilient to mistakes and miscommunication. Deceit, however, can eradicate cooperation, and emotional bookkeeping can make this even worse. We show that one possible defense against deceit is the evolution of a courtship process where animals can develop relationships without paying their full costs.

In many species, including most birds, humans, and various other animals, unrelated individuals form exclusive dyadic relationships with mutual attachment (1). Sensitively coordinated behavior is often critical to the success of these bonded pairs (2–5). Yet most of these species are not genetically monogamous (2), and individuals face conflicting interests. Behavioral coordination thus entails an iterated social dilemma: responding to a partner takes effort, which the partner might not reciprocate.

In addition to coordinated biparental care (6, 7), pair cooperation occurs across many other domains, such as mutual grooming (8), mate provisioning (9), social support (10, 11), consolation (12), nest building (13), and territorial and resource defense (14). Pair bonds can also occur without biparental care (1, 15), and diverse forms of cooperation often occur within the same pairs. Barnacle geese pairs, for example, collectively defend preferred foraging sites within colonies; males stand guard during foraging, allowing females to eat more, and males defend eggs while females take incubation breaks (16). Zebra finches negotiate incubation using vocal duets (17), and bonded pairs of marmosets facing novel social dilemmas overcame them by developing new forms of cooperation (18). All of these activities entail effort and attention between partners.

This is not to say that cooperation in bonded pairs is universal or unconditional: At evolutionary scales, investment in activities benefiting the partner must be traded off against investment in alternate sources of fitness, such as extrapair copulation and somatic maintenance. This tradeoff is evident, for example, in facultatively polygynous male starlings who reduced their incubation effort while increasing extrapair mating effort when their clutch size was reduced, leading to reduced hatching success as females did not compensate (19). Conversely, Gorman et al. (20) showed that zebra finch females paired with attractive males increased their share of incubation effort, and (16) observed that although bonded goose pairs had higher fitness, their longevity was not increased, indicating that parental effort was traded off against lifespan for both partners.

## Models of Pair Bonds.

Theories of sexual conflict have strongly influenced perspectives on biparental care (21). Early models of pair bonding viewed behavior through the lens of sexual selection in the “Battle of the Sexes” game, where male quality is equated with paternal care-giving that is signaled by courtship duration. Depending on costs, females in this game may evolve mixed or choosy (coy) strategies (22–27). In general, assuming an honest signal is observable, paternal care may evolve under sexual selection like other costly traits (28, 29). But since paternal care (male cooperation) occurs after mate choice (female cooperation), and can be modulated (30–32), it is not obvious why a premating signal of it should ever be honest. By treating paternal care as a fixed, observable trait, rather than an iterated strategic choice, these models sidestep the evolutionary problem of cooperation.

In contrast, McNamara et al. (33) represented biparental care as an iterative negotiation where each individual responds to the other’s previous move. This led to the prediction that under evolutionarily stable negotiation rules, individuals would partially compensate for changes in effort by their partners, resulting in inefficient offspring provisioning under biparental care. However, experimental manipulations have shown a range of responses from no response to full compensation to matching effort (34).

Relatively few models have explored pair bonding from the perspective of cooperation theory. Johnstone et al. (35) suggested that turn-taking could sustain cooperation. They extended the model of Houston (36) to allow for conditional cooperation, showing that reciprocity could ameliorate the inefficiency caused by sexual conflict if parents take turns to engage in parental care. Additionally, Barta et al. (37) showed that sex role division of labor could stabilize biparental care.

Song and Feldman (38) using analytical modeling and individual-based simulations showed that unconditional cooperation and pair bonding could coevolve in the snowdrift (also known as Chicken or Hawk-dove) game provided divorce rates were low and costs of pair bond maintenance did not exceed a threshold. This game is defined with the payoff matrix


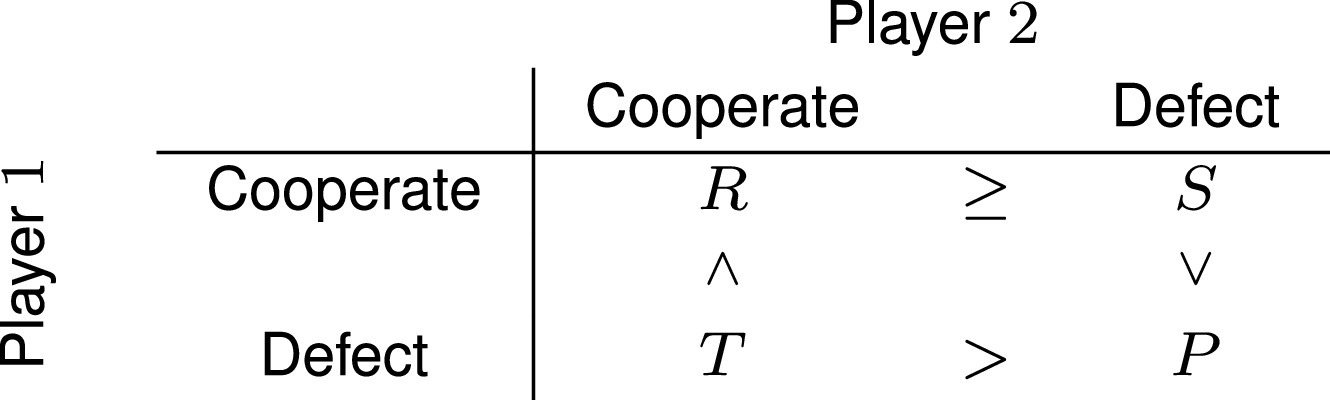


Snowdrift was chosen in that study to represent the benefit of biparental care in species where uniparental care is also rewarding. Successful reproduction requires costly care (outcomes R and S for biparental and uniparental care, respectively), but it is preferable that the care is given exclusively by the partner (outcome T). However, there is a catastrophic outcome if neither provides care (outcome P).

Cooperation dynamics are captured more generally by the prisoner’s dilemma (39). The prisoner’s dilemma payoff matrix is given by


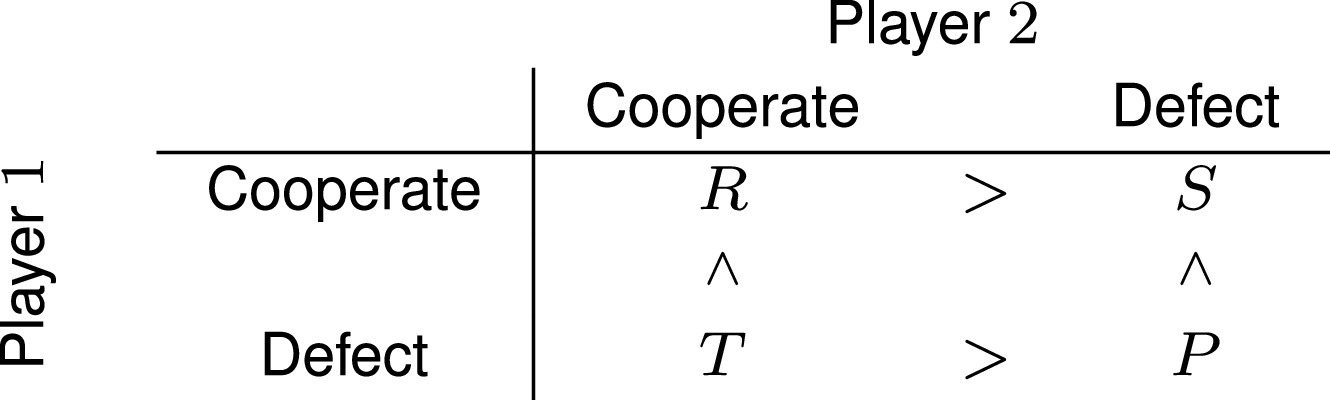


In contrast to snowdrift, in the prisoner’s dilemma, the sucker’s reward is smaller than the punishment for mutual defection, creating an incentive against unilateral cooperation (and thus a more challenging scenario for the evolution of cooperation). This can be interpreted as a scenario where the costs of uniparental care outweigh its benefits but is also applicable to numerous other social dilemmas where one-sided effort goes unrewarded. For example, social grooming requires effort which is rewarded only if reciprocated. Models suggest that for unrelated individuals, evolution and maintenance of cooperation in the iterated prisoner’s dilemma (IPD) is possible under restricted circumstances (40), such as when cooperators share honest signals (41), social networks allow for repeated interactions and conditional strategies such as Tit-For-Tat are allowed (42) or individuals can choose their partners (43). These conditions may be satisfied for bonded pairs, suggesting that cooperation could be theoretically plausible. Moreover, the observation of preferential association between cooperative partners has led to the notion of biological markets, where competition for preferred social partners may drive selection for honest signals of cooperation (44).

However, noise presents a major challenge to cooperation in the IPD (45). Neither senses nor actions are perfect, so cooperative relationships must be robust to both perceived and accidental harm. Popular reciprocity-based strategies such as Tit-For-Tat fail in the face of relatively limited noise (45, 46), and those that survive (such as generous or contrite Tit-For-Tat) tend to be exploitable and thus unstable (47). This presents an evolutionary puzzle because accurate detection of cooperative intent is likely a costly cognitive ability. Before the existence of cooperation, animals had no reason to evolve this costly trait, so accuracy likely evolved after cooperation. Johnstone and Savage (48) further developed the parental care model of ref. 35 to account for imperfect information, and found that turn-taking could be robust, in effect creating a form of reciprocity. However, this was true only when the costs and benefits of cooperation were time dependent, suggesting that it may be most relevant for pairs engaging in specific behaviors such as offspring provisioning rather than generalized cooperation.

Agents in an iterated social dilemma also have an incentive to deceive: that is, to influence their partner to perceive them as more cooperative than they are. Deceit arises frequently in competitive interactions within species as well as in interspecific interactions (49). However, outside large-brained primates (50, 51), empirical evidence for deceit between social partners remains scant (but see refs. 52 and 53). This could be because deceit within cooperative relationships is generally unfeasible, or because cooperation only survives when deceit is unfeasible.

Here, we model the evolution of strategic cooperation in pairs facing iterated social dilemmas in noisy environments with the possibility of deceit and varying divorce costs. Since we are interested in all forms of pair-based cooperation, not just biparental care, we use the IPD game with an infinite horizon (that is, the number of iterations is, in principle, unbounded). We explore the evolutionary role of two mechanisms by which pair-bonding species may resolve the problems of noise and deceit without resorting to complex cognition. These are emotional bookkeeping and courtship.

## Emotional Bookkeeping.

Strategic responses in relationships are often suggested to be constrained by costs of cognition and memory (54). Nonetheless, lasting social bonds occur in numerous species, with long-term reciprocity documented in several of these (55–57). For example, (58) and (59) showed that tufted capuchins and mandrills reciprocate grooming over long timeframes; likewise, flipper rubbing in bottlenose dolphins (60) and food sharing in vampire bats (61) are reciprocated long term.

Research suggests these social bonds may be mediated by affective mechanisms (56, 62), in particular emotional bookkeeping, a system where persistent relationship states (emotions or attitudes toward a specific partner) guide social decisions at minimal cognitive cost by integrating decision-relevant information across contexts and timescales (63–65). In this way, individuals may make adaptive social decisions with neither episodic memory encoding specific events nor foresight about future rewards (66).

Social neuroendocrine responses vary continuously, can be conditioned, and trigger behaviors that can create situations that stimulate further neuroendocrine response (67). For example, mammalian social grooming triggers endorphin (68, 69) and oxytocin release (70), increasing social memory formation (70, 71), which increases future grooming of the same partner (72). However, this positive feedback is highly sensitive to social context (70, 72), resulting in a dynamic equilibrium where future neuroendocrine responses and subsequent relationship behaviors can be adaptively fine-tuned in response to past interaction outcomes without complex strategic cognition. In this way, neuroendocrine-behavioral feedback provides a mechanistic basis for emotional bookkeeping (66). For example, (73) showed that oxytocin was elevated after grooming in socially bonded chimpanzees compared to nonbonded ones, suggesting that oxytocin tracks social bond strength.

Several models of emotional bookkeeping have been based on primate social networks and confirm that it can generate realistic behavior and long-term reciprocity in this context. Puga et al. (74) modeled bonding based on attachment driven by social grooming. When individuals preferentially interacted with those they groomed most often this led to increased reciprocity. Evers et al. (75) developed an agent-based model for macaque social behavior governed by 2-dimensional emotional bookkeeping (liking and fear) that successfully replicated a wide variety of empirical patterns; a follow-up model (76) integrated emotional responses to earlier grooming episodes via a “like” attitude and found that emotional bookkeeping over intermediate timescales enabled individuals to differentiate between partners, resulted in strongly reciprocated partner preferences that could be maintained over long periods.

Despite its likely role in long-term relationships, emotional bookkeeping has to our knowledge not previously been addressed in theoretical models of pair bonding. Insofar as pairs engage in iterated social dilemmas with partner choice across diverse timescales and scenarios, emotional bookkeeping may simplify the requisite social cognition. Mechanistically, vertebrates have converged across separate lineages to use the same neurochemical systems and neural circuitry in pair bonding, and these mechanisms strongly overlap with those of other social bonds (77).

Evidence suggests oxytocin, vasopressin, dopamine, and endorphin pathways contribute to pair bonding in humans, voles, mice, marmosets, and titi monkeys (78). In birds, mesotocin and arginine vasotocin may play a similar role (79), and homologous pathways are implicated in pair bonding fish (80, 81). Among humans, pair bonding is accompanied by release of dopamine, norepinephrine, and nerve growth factor along with serotonin depletion (82). These provide increased motivation and sensitize the individual to reward learning, resulting in escalating attachment. Simultaneously oxytocin and vasopressin strengthen social bonds, learning, and memory (10) while deactivating brain regions associated with negative emotions and social judgment (82) and increasing positive affect and cooperativity toward the partner (83).

## Courtship.

Social relationships often involve complex patterns of coordinated interactions between individuals. These include slightly beneficial but highly sensitive activities such as social play and mutual grooming in birds and mammals. Among pair-bonding birds and monogamous Old World primates, elaborate coordinated displays occur that have no obvious adaptive benefit (84). Such mutual displays are especially common in long-lived monomorphic species where sexual selection has little explanatory power (85).

Cooperative functions have been suggested to explain mutual displays, including shared territorial defense (84, 86, 87), signaling commitment within pairs (86, 88, 89), and coordinating breeding activities (90). Mate guarding has also been proposed as an explanation (84, 86), but empirical studies showing low levels of extrapair paternity in duetting species (91, 92) and insensitivity to conspecific abundance (92), bystander presence (93), divorce rates (92), or social monogamy (90) suggest otherwise. Another line of research suggests that displays by monogamous partners may sometimes be manipulative (94). For example, Servedio (85) showed that costly mutual displays within pairs could evolve if they stimulated increased parental investment by the displaying individual’s partner through a preexisting bias, provided that the increased investment by both parents had synergistic benefits to offspring. But why should mutual displays be coordinated within pairs?

Coordinated mutual displays are thought to provide advantages in shared territorial defense because display coordination provides an honest signal of coalition strength to outsiders (84, 87). However, several lines of evidence suggest that coordinated displays are at least equally important within pairs. Duet dancing in Java sparrows occurred in the absence of bystanders and was highly predictive of mating (93). Diniz et al. (91) found that in socially monogamous horneros, while duets were associated with territory quality, offspring survival was influenced by duets but not territory features. And in Neotropical wrens, duet coordination and consistency were greatest in species with long breeding seasons, but unrelated to intraspecific competition (92), again suggesting duet coordination is primarily relevant within pairs. Several authors (3, 5) have argued that interindividual coordination at behavioral and physiological levels underlies pair bond strength and successful reproduction across species. In humans, rhythmic interpersonal synchrony is linked to prosocial behavior (95, 96), especially when it involves fine-grained social coordination (97). Tapping or rocking in synchrony likewise generates trust (98) and affiliation (99).

Synchrony requires close attention, sensitivity, and mutual responsiveness between partners and therefore imposes an iterated series of small social dilemmas. Coordinated mutual displays typically develop at the beginning of relationships, before breeding activities commence and involve learning (2). For example, Rivera (89) found that plain wrens precisely coordinate their complex duets by dynamically adjusting their responses to their partners’ calls. Duet coordination involved pair-specific rules which were learned within the first few days of a new partnership (100). Similarly, in primates, siamang duets occur most frequently at the start of relationships (101), and their coordination improves with practice (102). Duetting in siamangs was positively correlated with behavioral synchrony, mutual grooming, and proximity between mates (103).

Roth et al. (5) proposed a positive feedback loop between interindividual coordination and pair bond strength, where mutual displays and other interactions during courtship and relationships reinforce long-term coordination. Similarly, Prior (3) suggests that behavioral coordination in brief social interactions contributes to parental coordination on larger timescales. Implicitly in these arguments, courtship interactions influence emotional bookkeeping, which suggests they should have neurophysiological effects.

This link was made more explicit by Savage et al. (104), who suggested that musicality functions in social bonding/affiliation among both humans and other animals. They noted that music simultaneously influences social preferences, coalition formation, identity fusion, and prosociality by creating long-lasting changes in group member affiliation and intragroup prosociality. These processes are, they argued, mediated by oxytocin and the endogenous opioid system which are implicated in social connection, affiliative and cooperative behavior as well as music and synchronized dancing. In humans, behavioral synchrony enhances endorphin release triggered by shared activities such as laughing, singing, dancing, feasting, and storytelling (105)—all activities that are common in courtship. Ulmer et al. (106) found that oxytocin mediated effects of endorphins and stress on behavioral synchrony.

Outside of humans, there have been limited studies of the neurochemistry of pair coordination, but available evidence is compatible with coordinated displays and interactions mediating emotional bookkeeping. Social grooming stimulates endorphin release in mammals generally and often precedes mating. Marmosets with strong social bonds also shared synchronized oxytocin fluctuations (107). Testosterone covaried within graylag goose pairs and this covariance was higher in more successful pairs (108). In great tits, corticosterone levels were similar within pairs, and their similarity increased with pair bond duration (109). Pairs whose corticosterone levels became more similar had greater reproductive success and were less likely to divorce.

Seen in the light of emotional bookkeeping, these observations suggest a unified adaptive function for courtship behaviors, including mutual displays, allogrooming, and other small coordinated interactions in pair bonds: Because they involve iterated social dilemmas, successfully coordinated courtship behaviors trigger neuroendocrine feedback that reinforces cooperation and attachment within pairs, leading to further improvement in coordination.

A strength of the emotional bookkeeping model is that different behaviors can influence the same bookkeeping variable, allowing individuals to integrate diverse sources of information about their relationships as seen in the multimodal mutual courtship displays of cordon-bleu finches (110). Thus many different activities—including ones that confer little or no direct benefit—can simultaneously strengthen cooperative pair bonds and act as an honest signal of relationship quality, allowing individuals to both evaluate and improve partnerships without risking the full cost of their partner defecting.

## Model

Modeling emotional bookkeeping requires that individuals have emotional state variables which they use to make decisions and update strategically based on their experiences. Because the benefit of emotional bookkeeping is thought to be its ability to integrate decision-relevant information across timescales, allowing evolution of both stochastic and deterministic strategies may be important, especially in noisy environments where uncertainty can be exploited. Allowing for courtship, divorce, and deceit adds further complexity.

Stability analysis often has limited utility in such scenarios because in discounted infinite horizon iterated games almost any payoff is attainable with a Nash equilibrium (111) (cf. ref. 112), and the complex dynamics of emotional bookkeeping likely preclude simplifications used in comprehensive analytical approaches. Consequently, in keeping with past work on the iterated prisoner’s dilemma and emotional bookkeeping (42, 74, 76, 113), we primarily used simulation. To explore the generality of our simulation results, we performed sensitivity analysis for model parameters, and dissected the model to examine the role of each component (emotional bookkeeping, courtship, divorce, and deceit) separately in a fully crossed experimental design. Our most reduced model corresponds to classical iterated prisoner’s dilemma models where strategic responses are based on a single-iteration memory (e.g. ref. 42). Finally, we developed an analytical approximation for further insight into the effects observed in simulation.

We used agent-based simulation to examine the evolution of cooperative pair bonds in agents who made decisions using up to four emotional states, $s=(s_{a},s_{c},s_{d},s_{p})$:

1. **attachment** ($s_{a}$): continue relationship (or end it)
2. **cooperativeness**$\left(s_{c}\right)$: cooperate (or defect)
3. **deceit**$\left(s_{d}\right)$: when defecting, appear to cooperate (or not)
4. **courtship**$\left(s_{p}\right)$: court (or pair bond)

Each of these states is a real value in the range [−1,1]. We simulated 500 agents evolving through a death-birth Moran process, with mortality rate m=0.01. At each iteration, dead agents were replaced by clonal offspring with mutation. Death was random, and agents were chosen for reproduction based on their lifetime payoff. Payoff was accrued by repeated prisoner’s dilemma interactions minus costs of divorce, courtship, and deceit. The model ran for 100,000 iterations.

**Fig. 1.**
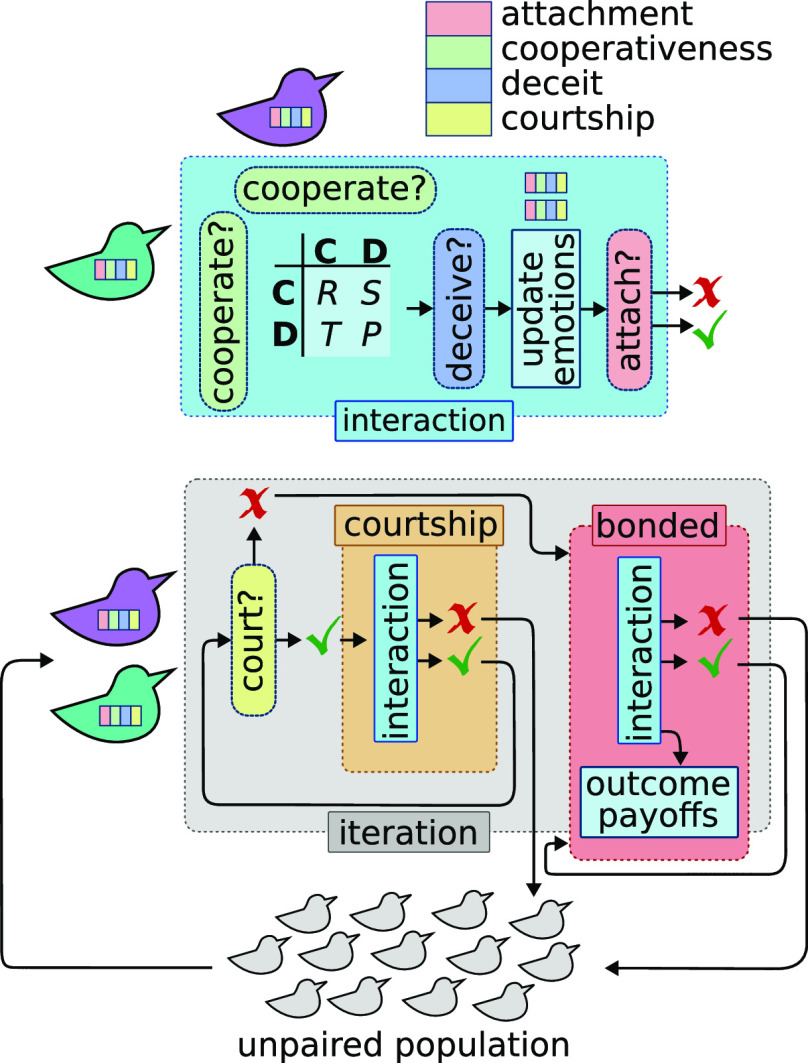
Schematic showing the main features of the model. Each iteration, a population of unpaired individuals is sampled for new pairings. Within an iteration (gray box), a new pair decides based on $s_{p}$ whether to court (orange box) or pair bond (pink box). Whether courting or bonded, they interact (blue box), choosing cooperate or defect based on $s_{c}$ possibly with deceit based on $s_{d}$ and update their emotions. Bonded pairs receive a prisoner’s dilemma payoff from the interaction, whereas courting pairs do not. As the last stage of the interaction, pairs decide whether to remain attached using $s_{a}$. If unattached, they return to the unpaired population and the iteration ends. Otherwise, if courting, they choose whether to court again; if pair bonded, the iteration ends and they will remain bonded in the next iteration.

Fig. 1 illustrates the key processes of the model. Each iteration, all unattached agents were paired at random and chose whether to court or pair bond. During courtship, they could end the relationship without cost; after each courtship interaction, they again chose whether to court or bond, for up to 20 courtship interactions within one iteration. Analysis of the actual number of courtship interactions (top quartile average of 2.94, max average of 10.48; *SI Appendix*, Fig. S1 and Table S1), shows that in practice this limit did not constrain evolution. Pairs that chose to bond then played a single prisoner’s dilemma round as a bonded pair within the same iteration; otherwise, they remained unattached, forfeiting the payoff for that iteration. Courting and ending relationships were unilateral choices; pair bonding and continuing relationships required consensus. Bonded pairs played one prisoner’s dilemma per iteration with payoffs R= 3,T=5,S=-1, *P* = 0. Pair bonds continued so long as both partners survived and neither chose to end the relationship; if either chose to end the relationship, both paid a divorce cost $c_{\mathrm{div}}$ and were unattached in the next iteration.

### Decisions.

Agents made binary decisions using relevant emotional states ($-1<s_{e}<1$, e∈{a,c,d,p}). The emotional state is mapped to a probabilistic binary choice with a piece-wise linear sigmoid function

$$\pi \left(s\right)=\left\{\begin{array}{cc} 0 & \;\text{if}s<-\frac{1}{2}\\ s+\frac{1}{2} & \;\text{if}-\frac{1}{2}\leq s<\frac{1}{2}\\ 1 & \;\text{if}\frac{1}{2}\leq s \end{array}\right..$$

Thus, decisions were linearly probabilistic when ($-0.5\leq s_{e}<0.5$) and deterministic outside this range. The piecewise linear function is commonly used in decision models as a computationally efficient approximation of the smooth sigmoid 1/(1+exp(−a∗x)) (114, 115). In these models, the sigmoid or softmax transformation from policies (in our case, emotional states) to action probabilities allows emergence of strategies ranging from highly deterministic to highly stochastic (whereas directly using policy values as action probabilities would make the strategy space overwhelmingly stochastic) (see, e.g., ref. 116). Comparison runs using a smooth sigmoid confirmed that the piecewise linear function did not impact results (*SI Appendix*, Fig. S2*A*).

### Genetics of Emotion.

Emotional state updates were regulated by genetic traits. For unattached agents, each emotion was set to a genetically determined base value when interacting with a new partner. So long as two agents interacted (either courting or as a bonded pair), their emotions were updated each interaction based on genetically determined responses to the interaction’s outcome. Emotions within relationships decayed toward the baseline at a genetically determined rate.

Agents had 24 genetic traits in total, with 6 traits for each of the 4 emotional states. That is, for emotional state e∈{a,c,d,p}, we had trait values for the base $G_{e}^{b}$, the decay rate $G_{e}^{v}$, and one trait for each prisoner’s dilemma outcome $G_{e}^{R},G_{e}^{T},G_{e}^{P},G_{e}^{S}$. Separate traits encoding the base value and response to each outcome are needed to represent key strategies like Win–Stay/Lose–Shift. The decay rate prevents emotional bookkeeping variables becoming extreme whenever the same outcome was experienced repeatedly (which would make stochastic strategies impossible). Fixing values for the decay rate would also constrain the evolvable strategy space; therefore, we made it an evolving trait as well.

Initial trait values were sampled from a Gaussian distribution with parameters μ=0 and σ=0.25. Mutation per trait was also Gaussian, but with σ=0.04. Trait values were constrained to $-1<G_{e}^{x}<1$ (for x∈{b,v,R,T,P,S}). Emotions were modified after each prisoner’s dilemma interaction t as:

[1] $$s_{e}\left(t+1\right)={\hat{s}}_{e}\left(t\right)+\left(G_{e}^{b}-{\hat{s}}_{e}\left(t\right)\right)\cdot \left|G_{e}^{v}\right|,$$

where ${\hat{s}}_{e}\left(t\right)=s_{e}\left(t\right)+\left(2\cdot G_{e}^{\Omega }\right)$ is the raw effect of the outcome Ω∈{R,T,P,S}, before decay, on emotions. When the iteration number is irrelevant or clear from context, we will simply write $s_{e}$.

### Courtship.

Courtship was represented as a series of instantaneous interactions between prospective partners that altered their emotional states but not their payoff (other than a fixed interaction cost per round). Choices in courtship interactions were governed by the same emotional architecture as the prisoner’s dilemmas between bonded pairs, with emotional states updated in the same way. However, courtship outcomes did not affect payoff. Each courtship interaction carried a fixed cost $c_{\mathrm{cou}}$ to both partners.

### Noise.

Here, we primarily studied the effects of perceptual noise, in which an individual acts as intended, i.e., both partners receive the appropriate payoff, but their partner responds as though they performed the opposite action. In our model, each emotional state was modified by $G_{e}^{R}$ for S, $G_{e}^{S}$ for R, $G_{e}^{T}$ for R and $G_{e}^{P}$ for T with probability parameter ξ.

We also considered action noise, in which an individual performs the opposite action of their intention. This is sometimes termed “trembling hand” in game theory. Action noise in the model was represented by changing an individual’s prisoner’s dilemma action (cooperate or defect) to the opposite action with probability parameter ξ.

### Deceit.

A defecting agent could at some cost appear to cooperate, i.e., their partner’s emotional states were modified by $G_{e}^{R}$ for S and $G_{e}^{T}$ for P. We represented deceit as a choice after defection based on emotional state $\left(s_{d}\right)$ with a fixed cost $c_{\mathrm{dec}}$ per deception.

### Experiments.

To understand the effect of emotional bookkeeping, courtship, deceit, and divorce, we tested our model with and without these features.

When emotional bookkeeping was disabled, agent emotion was set during each interaction to the genetically determined trait values for that interaction’s outcome (i.e., $s_{e}=G_{e}^{R},G_{e}^{S},G_{e}^{T},$ or $G_{e}^{P}$, as appropriate). This allows the evolution of strategies such as Tit-For-Tat that use a single-interaction memory, as seen in classical iterated prisoner’s dilemma models, but prevents the integration of information across timescales that occurs in emotional bookkeeping.

In the no-courtship condition, newly paired agents bonded immediately with no possibility of courtship. Similarly, in the no-deceit condition, there was no deceit. In each of these cases, the genetic architecture for the relevant emotional state was left intact but nonfunctional, i.e., drifting; this was done on the principle that minimal changes to different experimental conditions provide maximal code readability and ease of replication. In the no-divorce condition, bonded pairs remained bonded until either partner died (however, courting agents could end their relationships based on their attachment state).

In a fully crossed design, we varied the noise level (ξ= 0.0, 0.04, 0.08, 0.12, 0.16, 0.20) and type (perceptual versus action noise) and costs of courtship ($c_{\mathrm{cou}}$= 0.0, 0.4, 0.8), divorce ($c_{\mathrm{div}}$= 0.0, 4.0, 16.0) and deceit ($c_{\mathrm{dec}}$= 0.0, 0.4, 0.8, 1.2, 1.6) over 112 replicates. See ref. 117 for access to simulation code and data, analysis of sensitivity to additional model parameters (population size, mortality, mutation rate, initial genotypes and variance) (*SI Appendix*, Fig. S3), and behavior under staghunt (R=3, T=1, S=−1, P=1) and snowdrift (R=1,T=3,S=1,P=−1) payoffs (*SI Appendix*, Fig. S2). Varying the payoff matrix within the prisoner’s dilemma is not expected to lead to qualitatively different results (118, 119).

## Results

**Fig. 2.**
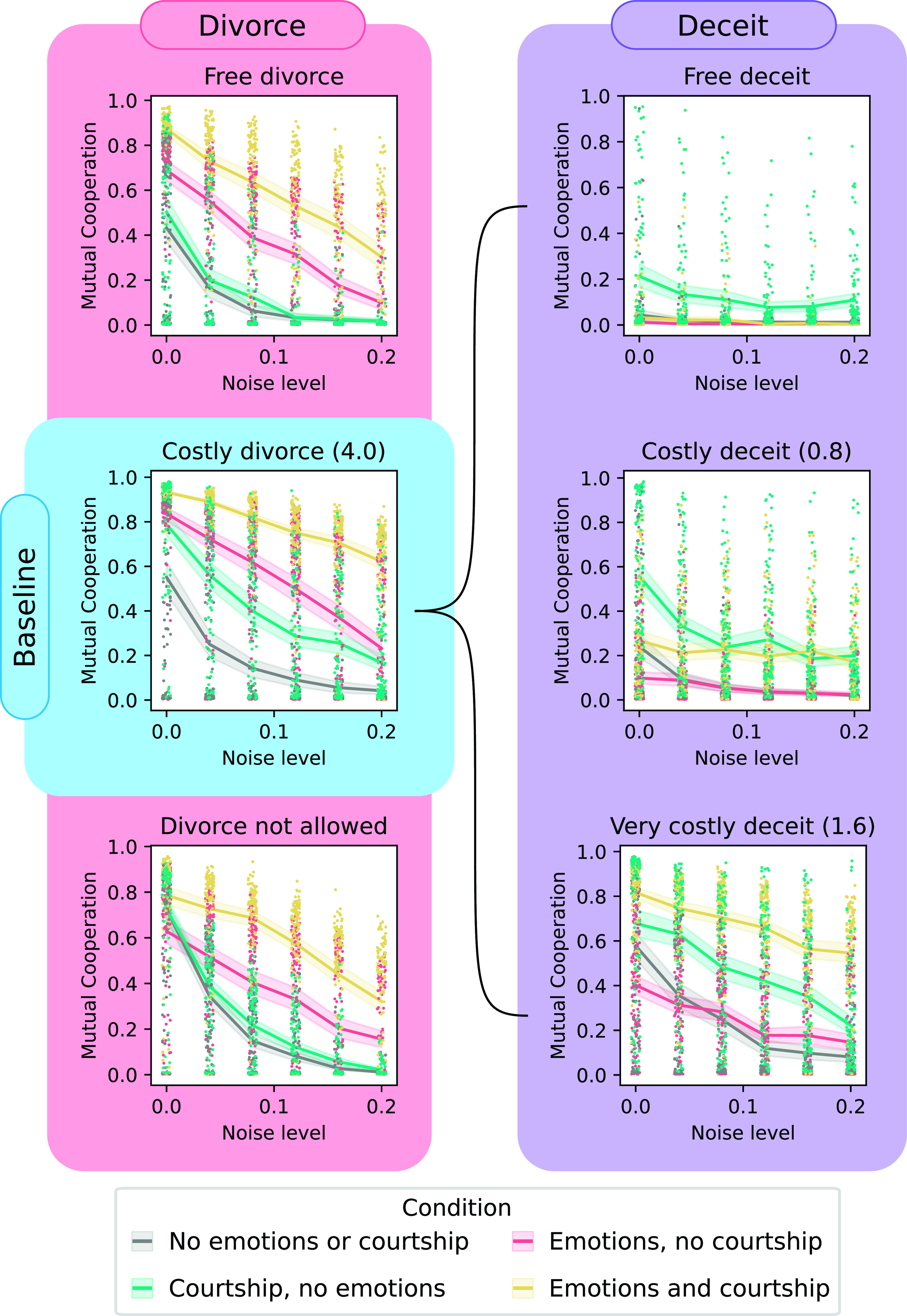
Mutual cooperation rate with and without emotional bookkeeping and courtship graphed against perceptual noise level. The baseline scenario shown here (*Center Left*) has costly divorce and no deceit ($m=0.01,c_{\mathrm{div}}=4.0,c_{\mathrm{cou}}=0.0$) and is compared against scenarios with free divorce (*Upper Left*), no divorce allowed (*Lower Left*), and deceit (*Right* column) with varying costs $c_{\mathrm{dec}}=0.0,0.8,1.6$ from *Top* to *Bottom*. Markers show results from individual runs. Lines show mean with 95% CI shaded.

Results from the fully crossed design are presented in *SI Appendix*, Figs. S4–S9. Here, we focus on scenarios with perceptual noise and no courtship cost ($c_{\mathrm{cou}}=0.0$; Fig. 2). Other scenarios led to similar patterns to the ones reported here in detail, with lower overall cooperation under action noise, and courtship costs modulating the evolutionary impact of courtship. In many conditions mutual cooperation levels were extremely variable between replicates, suggesting dynamics were dominated by critical transitions as is commonly observed in models of evolution of cooperation in the prisoner’s dilemma (e.g., ref. 120), and especially in the context of noise (121). Nonetheless, experimental conditions led to clear differences in average cooperation.

We first consider a baseline scenario (Fig. 2, *Center Left* panel in blue), with moderate divorce costs and no deceit. In this scenario, emotional bookkeeping and courtship each increased the noise resilience of cooperation and worked synergistically when combined. The effect of emotional bookkeeping was generally stronger than that of courtship. Sensitivity analysis (*SI Appendix*, Fig. S3) showed that emotional bookkeeping and courtship independently increased cooperation across broad conditions, and the synergistic benefit of combining them persisted across noise types, mutation and mortality rates, initial genotypes and variance, and in larger populations. This synergistic benefit was also seen in experiments with staghunt and snowdrift payoffs (*SI Appendix*, Fig. S2). In staghunt, cooperation was generally higher and courtship alone had little impact or decreased cooperation in these scenarios. In snowdrift, combining emotional bookkeeping and courtship increased social welfare the traditional metric of success in this game (122).

### Effect of Divorce.

At low noise levels without courtship, deceit, or emotional bookkeeping, prohibiting divorce led to greatest cooperation (Fig. 2, *Left* panels). However, in most other conditions, cooperation was greatest when divorce was possible but costly. Courtship had a large effect when divorce was costly but was only slightly effective when divorce was free or prohibited. In contrast, the effect of emotional bookkeeping was not strongly altered by divorce condition.

### Deceit Resilience.

Free or low-cost deceit destroyed cooperation under most conditions, and emotional bookkeeping often exacerbated this effect (Fig. 2, *Right* panels). Courtship however somewhat increased cooperation, and when deceit was highly costly, the combination of courtship and emotional bookkeeping restored cooperation.

### Strategy Evolution.

Analysis of the final genotypes of the population revealed a variety of evolved strategies, most of which were reminiscent of known ones. Since agents have multiple choices in our model and may change their decisions based on emotional state as well as interaction outcomes, it is not strictly possible to classify their strategies according to traditional criteria. Instead, we classified strategies as shown in Table 1 according to whether they would likely divorce, cooperate, or defect after interaction outcomes R,T,P, and S. This was calculated using the formula in Eq. **1**, which gives an updated emotional state $s_{e}^{\prime }$ based on a prior emotional state $s_{e}$, the relevant genetic traits $G_{e}$, and an outcome O∈R,T,P,S. We say an individual “would likely divorce” if $s_{a}^{\prime }\leq 0$, “would likely cooperate” if $s_{a}^{\prime }>0$ and $s_{c}^{\prime }>0$ and “would likely defect” otherwise. Since agents who divorce do not interact again, their cooperativeness is irrelevant.

**Table 1.** Strategy classification

	Action on outcome				
Strategy	R	T	P	S	Interpretation
Cooperator	Coop	Coop	Coop	Coop	Always cooperates
No S	Coop	Coop	Coop	Div/Def	Cooperates except on S
Reciprocator	Coop	Coop	Div/Def	Div/Def	Similar to Tit-For-Tat
Generous Reciprocator	Coop	Coop	Div/Def	Coop	Similar to Generous Tit-For-T
WSLS-like	Coop	Div/Def	Coop	Div/Def	Exploits T but cooperates after retaliation
Generous WSLS-like	Coop	Div/Def	Coop	Coop	WSLS-like, but cooperates on S
Trigger	Coop	Div/Def	Div/Def	Div/Def	Cooperates only after mutual cooperation (R)
Generous Trigger	Coop	Div/Def	Div/Def	Coop	Like Trigger, but cooperates on S
Opportunist	Def	Coop on at least one	Div/Def	Opportunistic defector
Generous Opportunist	Def	Coop on at least one	Coop	Like Opportunist, but cooperates on S
Quitter	Div	Coop on at least one	any	Mutual cooperation leads to divorce
Defector	Div/Def	Div/Def	Div/Def	any	No cooperation (receives S only due to noise,
					so S response drifts)

**Fig. 3.**
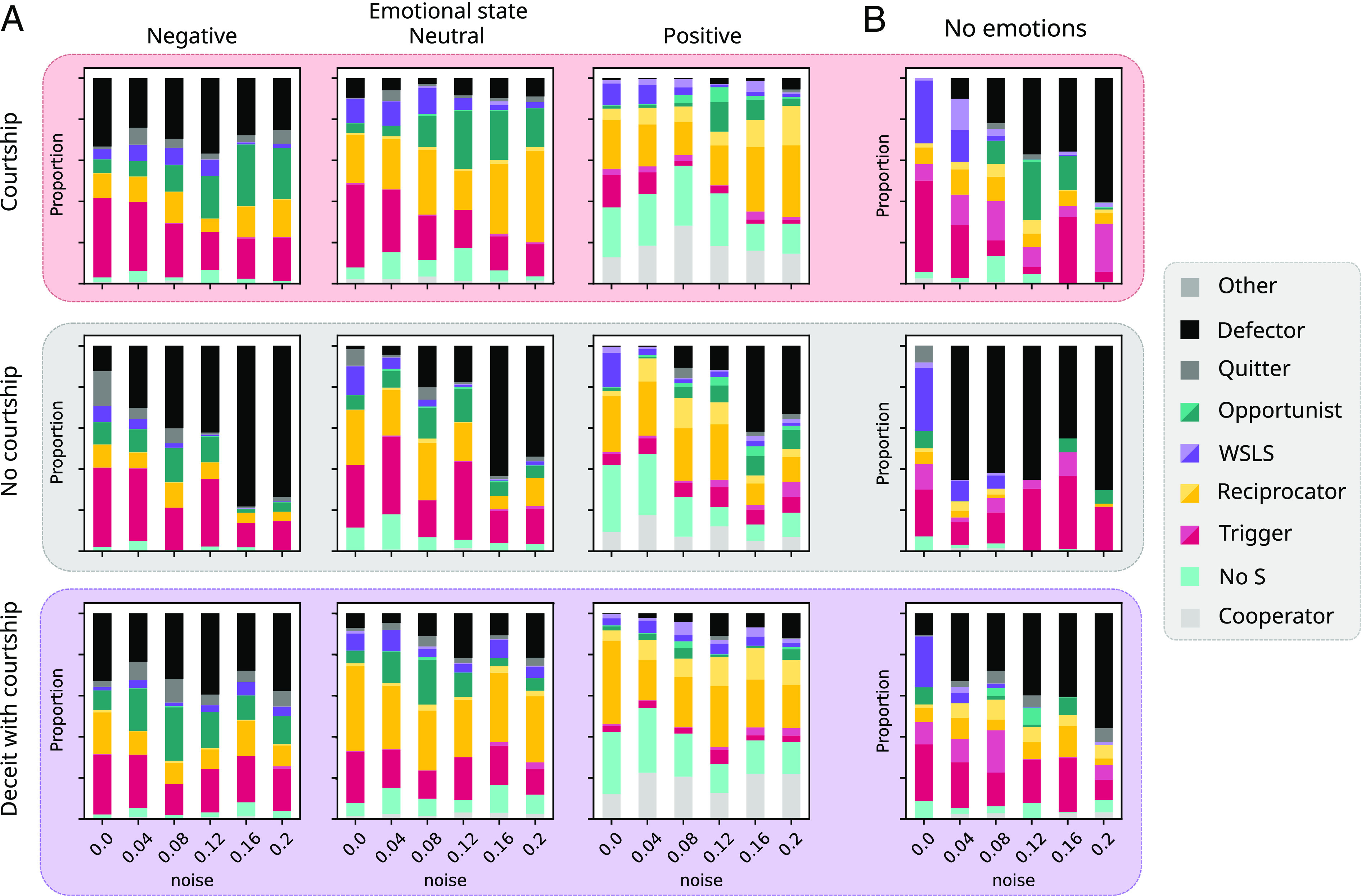
Evolved strategies as seen in the last iteration over 12 replicas in scenarios with and without courtship, emotional bookkeeping, and deceit (parameters $m=0.01,c_{\mathrm{div}}=4.0,c_{\mathrm{cou}}=0.0,c_{\mathrm{dec}}=1.6$). Strategies are classified as described in Table 1. Shading indicates whether strategies were “generous,” i.e., their response on S (light = cooperate, dark = divorce or defect). (*A*) Strategies in scenarios with emotional bookkeeping, by prior emotional state (negative: $s_{c}=-1,s_{a}=-0.5$; neutral: $s_{c}=0,s_{a}=0$; positive: $s_{c}=1,s_{a}=1$) (*B*) Strategies in scenarios without emotional bookkeeping.

We classify strategies where prior emotional states were negative ($s_{c}=-1,s_{a}=-0.5$), neutral ($s_{c}=0,s_{a}=0$), and positive ($s_{c}=1,s_{a}=1$) (Fig. 3*A*). We use $s_{a}=-0.5$ for the negative state of attachment because individuals with lower attachment than this would have deterministically divorced in the previous iteration, making subsequent relationship choices impossible. Notably, under this classification, the same agent may use different strategies depending on its emotional state.

For example, Win–Stay/Lose–Shift (WSLS) responds with defection to T and S, and cooperation to R and P. An agent with neutral prior emotional state ($s_{c}=0,s_{a}=0$) and genotype $G_{c}^{R},G_{c}^{P}=1$, and $G_{c}^{T},G_{c}^{S}=-1$ along with $G_{a}^{R},G_{a}^{S},G_{a}^{T},G_{a}^{P}=1$, and $G_{a}^{v}=0$ implements this strategy. A similar agent that has $G_{a}^{S}=-1$ would divorce on S regardless of $G_{c}^{S}$; we class this as WSLS-like as well. However, a strategy with the same $G_{c}$ values, but $G_{a}^{T},G_{a}^{P}=-1$ would be categorized as Trigger because it would divorce on T and P.

Fig. 3 shows the prevalence of different strategies in 12 replicates under different conditions, for scenarios with (Fig. 3*A*) and without emotional bookkeeping (Fig. 3*B*).

First, we consider scenarios without emotional bookkeeping (Fig. 3*B*). In this case, defectors and triggers were common in noisy scenarios, with WSLS-like strategies also frequent at low noise levels. Reciprocators and sometimes cooperators occurred as minority strategies. In some conditions, we also observed opportunists, who would defect on R but cooperate on T and/or P. Cooperative strategies often took generous forms, i.e., cooperating on S.

For agents with emotional bookkeeping (Fig. 3*A*), strategic responses changed with their prior emotional state. At positive prior emotion ($s_{c}=1,s_{a}=1$), a large proportion of agents acted as cooperators or reciprocators, or would cooperate with any outcome except S (No S). Defectors were frequent only at high noise levels, and WSLS-like, trigger, and opportunist strategies occurred in low proportions. Strategies in these scenarios were often generous on S, especially at higher noise levels. However, at neutral prior emotion ($s_{c}=0,s_{a}=0$), cooperators, and No S became rare and trigger and defector strategies increased. When prior emotion was negative ($s_{c}=-1,s_{a}=-0.5$), reciprocity also declined, with cooperation dominated by triggers and opportunists. Cooperative strategies at ($s_{c},s_{a}<=0$) rarely forgave S regardless of noise level, and a significant minority of agents became quitters who would divorce upon mutual cooperation even if they would cooperate on other outcomes, suggesting the relationship is likely irrecoverable from this emotional state.

Multivariate ANOVA confirmed that prevalence of strategies differed between conditions for courtship, deceit, emotional bookkeeping, and noise levels (P<0.001) (details in *SI Appendix*, Tables S2–S4). Follow-up comparisons showed that courtship increased No S (P<0.05) and cooperator strategies (P<0.001) while decreasing defectors (P<0.001). Emotional bookkeeping reduced the relative frequency of generous opportunists (P<0.05) and generous reciprocators (P<0.001). Deceit only influenced defectors (P<0.05), while higher noise decreased WSLS (P<0.05) and No S (P<0.05), but increased defectors (P<0.001). In scenarios with emotional bookkeeping, prior emotional state was important for all strategies (P<0.05) except WSLS and opportunists, with positive emotional state favoring generous and cooperative strategies while negative states increased quitters and defectors.

### Analytical Approximation.

The simulation results above show synergistic benefits of emotional bookkeeping and courtship across a wide range of conditions. To better understand this synergy, we developed an analytical model. Here, we consider a focal individual’s update to emotional state $s_{e}$ as given by Eq. **1**. For simplicity, here, we assume that neither the genotype values nor the emotional states are bounded, except for the decay $G_{e}^{v}$ which is nonnegative. We also only consider perceptual noise with rate ξ. The expected updated value of an emotional state $s_{e}$ after an interaction can then be written as

[2] $$\begin{array}{c} \begin{array}{cc} s_{e}\left(t+1\right) & =s_{e}\left(t\right)\left(1-G_{e}^{v}\right)+G_{e}^{b}G_{e}^{v}\\ & \;+\vec{\omega }\left(t\right)\cdot {\left[2\left(1-G_{e}^{v}\right)\left(\left(1-\xi \right)G_{e}^{i}+\xi G_{e}^{j}\right)\right]}_{\left(i,j\right)}, \end{array} \end{array}$$

where $\vec{\omega }$ is the vector of outcome probabilities (at interaction t)

$$\begin{array}{cc} \vec{\omega }\left(t\right) & :=\left[\begin{array}{c} \pi \left(s_{c}\left(t\right)\right)\pi \left(s_{c}^{\prime }\left(t\right)\right)\\ \pi \left(s_{c}\left(t\right)\right)\left(1-\pi \left(s_{c}^{\prime }\left(t\right)\right)\right)\\ \left(1-\pi \left(s_{c}\left(t\right)\right)\right)\pi \left(s_{c}^{\prime }\left(t\right)\right)\\ \left(1-\pi \left(s_{c}\left(t\right)\right)\right)\left(1-\pi \left(s_{c}^{\prime }\left(t\right)\right)\right), \end{array}\right] \end{array}$$

for two individuals with cooperation state $s_{c}\left(t\right)$ and $s_{c}^{\prime }\left(t\right)$ respectively; and i runs over outcomes {R,S,T,P}, and j over flipped outcomes by noise {S,R,P,T}.

If we assume that the decay is trivially small (i.e., $G_{e}^{v}\approx 0$), then the Eq. **2** simplifies to

$$s_{e}\left(t+1\right)=s_{e}\left(t\right)+2\vec{\omega }\left(t\right)\cdot \left(\left(1-\xi \right)G_{e}^{i}+\xi G_{e}^{j}\right).$$

As a consequence, whether an emotional state increases or decreases in value depends only on the sign of the term $\vec{\omega }\cdot \left(\left(1-\xi \right)G_{e}^{i}+\xi G_{e}^{j}\right)$, which intuitively represents the emotional response weighted by the probability of the outcome and the possibility of noise. We will refer to this term as $\Delta s_{e}$ (that is, $s_{e}\left(t+1\right)-s_{e}\left(t\right)$).

We argue that emotional bookkeeping is useful to detect stochastic defectors in noisy environments. To see this, notice that if the focal individual cooperates deterministically ($\pi (s_{c}\left(t\right))=1$) and their partner cooperates with fixed probability $x(=\pi \left(s_{c}^{\prime }\left(t\right)\right))$, then

$$\begin{array}{cc} \Delta s_{e} & =x\left(\left(1-\xi \right)G_{e}^{R}+\xi G_{e}^{S}\right)+\left(1-x\right)\left(\left(1-\xi \right)G_{e}^{S}+\xi G_{e}^{R}\right)\\ & =G_{e}^{R}\left((1-\xi )x+(1-x)\xi \right)+G_{e}^{S}\left(\xi x+(1-x)(1-\xi )\right), \end{array}$$

which is monotonic in x.

Then, so long as $\xi <\frac{1}{2}$, the focal individual can set a threshold ϵ>0 and values for their attachment genes $G_{a}^{b}>0$, $G_{a}^{R}>0$, and $G_{a}^{S}<0$ such that the attachment is positive if and only if x>1−ϵ. This strategy can be used to divorce partners that are trying to take advantage of noise to stochastically defect while remaining undetected. This detection strategy is not possible without emotional bookkeeping. Moreover, the usefulness of this strategy is enhanced by the presence of courtship, as the cost of courtship can then be offset by the benefit of identifying stochastic defectors and ending those relationships during courtship at reduced cost.

## Discussion

Our results show that emotional bookkeeping allows the evolution of noise-resilient cooperative strategies for the prisoner’s dilemma within bonded pairs, linking the extensive evidence for neuroendocrine-behavioral feedback underlying pair bonding (78, 123, 124) to the benefits of intrapair cooperation (2–5). The combination of emotional bookkeeping and courtship especially increases cooperation in pair bonds where communication is noisy and divorce is costly. Our analytical model suggests that this synergistic benefit arises because emotional bookkeeping during courtship allows early detection of stochastic defectors which would not otherwise be possible in noisy environments.

### Courtship.

Our simulation findings support the view that coordinated courtship interactions, by triggering pair bonding neuroendocrine feedback, may provide a mechanism for honest evaluation of relationship quality and in this way support the evolution and maintenance of cooperation in bonded pairs, as argued by Roth et al. (5).

From the perspective of our model, courtship includes any interaction that succeeds only through mutual investment and has little intrinsic fitness consequence, encompassing both the elaborate duets and dances which are common in pair bonding birds and primates (84, 86), as well as more subtle interactions—small talk, flirtation and social play. From a human perspective, we do not normally think of these behaviors as costly because we are intrinsically motivated to engage in them. However, from an evolutionary perspective, it is precisely this intrinsic motivation to perform a cognitively, temporally, and energetically demanding activity that requires an adaptive explanation. With theory over recent decades largely focused on the role of displays in sexual selection and sexual conflict, there has been little appetite for empirical studies of subtle cooperative interactions in pair bond development (125). Our results suggest that a renewed focus on this area is warranted.

Early ethologists were convinced that the flamboyant displays of new pairs were preceded by less costly reciprocal interactions. For example, Huxley (126) in his classic study of great crested grebe courtship says he was unable to observe the pairing-up process, but “the keeper tells me there is much flying and chasing about... From analogy with other birds and with ourselves we should expect that the chasing was the expression of felt but unreasoned likes and dislikes, and that the courtship-actions were only gone through after the two birds had become fairly well-disposed toward each other.” Lorenz (127) likewise noted that in graylag geese, pair bonds often develop gradually out of gosling friendships, and begin with incipient pairs walking in synchrony and making frequent eye contact. Such observations have been widely criticized for their subjective content, and until recently hypotheses about subtle social interactions occurring in natural contexts were difficult to test rigorously. However, automated analysis of video footage with machine learning offers opportunities for ethological studies of these behaviors at larger scales and without the biases imposed by human observers (128–130).

### Emotions and Deceit.

We argue that the benefits of cooperative pair bonds are most likely gleaned through emotional bookkeeping. Populations that have not cooperated in the past have no reason to pre-evolve the cognitive architecture to accurately recognize cooperative intent. Therefore, these populations would likely initially experience high levels of perceptual noise. Our results show that in this situation, emotional bookkeeping greatly facilitates the origin of cooperation.

However, we also found that bonds relying on emotional bookkeeping were highly vulnerable to deceit. Deceit may become increasingly likely as sophisticated social cognition evolves in socially bonding species due to Machiavellian selection (131). Courtship could sometimes prevent the deceit-driven collapse of cooperation provided deceit remained highly costly, but it is not clear how common this is. In general, we find that cooperation will persist only in contexts where deceit remains unfeasible or extremely expensive.

Evidence for relationships between complexity in cognition and social structure remains ambiguous (131). Aureli et al. (62) suggest that one reason for this may be that emotional bookkeeping constrains decision-making, allowing animals to navigate social complexity without sophisticated cognition, at costs in behavioral flexibility. Our results suggest that one such cost may be the collapse of cooperation when deceit evolves. This may be why, despite tantalizing suggestions of Machiavellian cognitive evolution in other species (132), we have found few reports of deceit within bonded pairs outside of humans (53, 133).

This observation may also explain the rarity of genetic monogamy in otherwise highly cooperative pairs (134). Defections on shared activities such as social grooming, incubation, nest building, and territorial defense are likely to be difficult or impossible to conceal, but deceit in sexual fidelity may often be feasible at low cost; if so, cooperation on this aspect is unlikely to evolve or persist.

### Divorce.

The role of divorce in pair cooperation remains controversial: some models emphasize that long relationship duration is critical for high cumulative payoffs from cooperation in iterated games (38), but others suggest that in real-world conditions, the benefit of flexible social bonds may outweigh that of relationship duration (135, 136). Cooperation in our model was often greatest with moderate divorce costs. Under a range of conditions, cooperators were common and would usually divorce defectors but not fellow cooperators. In these scenarios defectors experienced much more frequent costly divorces than cooperators, making divorce an effective collective punishment for defection. This finding recalls Bergstrom et al.’s (137) observation that courtship may function as mechanism design by altering the incentive structure faced by individuals in ways that favor cooperation. They showed that cooperation in the iterated Dictator game could be stabilized by enforcing a time-wasting courtship period. For real animals, divorce is nearly always possible, but divorce is likely costly in terms of mate replacement and pair bond development (2). Our model highlights that these natural conditions may be relatively favorable for pair cooperation.

### Strategies.

Populations in our model evolved strategies that approximate ones found to be successful in both theoretical models (reciprocity, Win–Stay/Lose–Shift, All-D) and widely used by humans (reciprocity, trigger, All-D) (138). In scenarios with emotional bookkeeping, both conditional and unconditional cooperation were common, with generous and unconditional strategies occurring mainly in positive emotional states. The most prevalent conditional cooperative strategies were triggers and reciprocators. Unconditional cooperation was rare in scenarios without emotional bookkeeping, but conditional strategies in these scenarios were often generous. Strategies resembling Win–Stay/Lose–Shift were common at low noise levels.

A frequently observed minority strategy was to respond with cooperation to T and/or P, while defecting after R. We term this strategy “opportunist” because it exploits unconditional and generous cooperative strategies. In the context of the model, a reciprocator or trigger strategy might not give an instantaneous or deterministic negative response to defection, allowing opportunists to fine-tune occasional defections against generous variants of these dominant strategies. Opportunists occurred in scenarios with and without emotional bookkeeping. Although our analytical model suggests that emotions and courtship allow cooperators to detect opportunists, this did not lead to decreased opportunism. This may be because the increased frequency of cooperative strategies in these scenarios counterbalanced any decline in individual exploitability, such that opportunism remained an attractive strategy.

### Conclusions.

The occurrence of diverse forms of cooperative behavior within bonded pairs presents a puzzle that theories based in sexual conflict and parental investment have struggled to explain. We have shown that two key features of vertebrate social bonds—emotional bookkeeping and courtship behavior—facilitate the evolution of robust cooperation in pairs playing the iterated Prisoner’s dilemma.

Griffith (2) proposed four questions for future research on cooperative pair bonds. “What is the value of a good partnership to evolutionary fitness? How is a good partnership made? What are the traits that contribute toward good partnerships? What are the ecological or evolutionary drivers that favor good partnerships?” Our models suggest some partial answers and raise further questions.

Good partnerships create resilient mutual cooperation that can resist errors and incentives for defection, such that pairs accrue long-term fitness gains. They can be forged through a combination of iterative courtship interactions with emotional bookkeeping, which together allow individuals to gather and integrate information about their relationships at low cost, thus permitting the evolution of cooperation that is resilient to noise. In particular, courtship with emotional bookkeeping allows detection and divorce of opportunistic defectors.

Traits that contribute to good partnerships depend on the surrounding population. In our model, accepting only mutual cooperation as an outcome of interactions emerged as a robust cooperative strategy, and reciprocity also evolved in a wide range of conditions. Among agents using emotional bookkeeping, unconditional cooperation also flourished, but only when agents’ emotional states were positive (i.e., strongly cooperative and attached to their partners). We found that the evolutionary drivers of good partnerships included low noise, cheap courtship, costly divorces, and deceit being unfeasible or extremely expensive.

Do these findings also apply to relationships other than pair bonds? Some observations suggest they might: Coalitions of male chimpanzees, for example, are initially built via low-cost social grooming and later extend to social support in potentially costly dominance challenges. In larger groups, coordinated displays are also associated with collective cooperation (87). However, strong cooperation with low relatedness remains rare outside pair bonds, suggesting that social network structure may constrain cooperation even with emotional bookkeeping and courtship.

The prisoner’s dilemma creates a restrictive scenario for the evolution of cooperation. For naturally occurring pairs, a wide range of possible actions and payoffs might be expected due to the varying forms of cooperation possible within pairs. Future work that takes into account the diversity of possible interactions within pairs as well as broader social network structures could give further insight into these issues.

## Supplementary Material

Appendix 01 (PDF)

## Data Availability

Colab notebook with code, dataframes with experimental results and analysis data have been deposited in Github repository (117).
