# Supplementary material for: Emotions and courtship help bonded pairs cooperate, but emotional agents are vulnerable to deceit
Source: Proc Natl Acad Sci U S A. 2023 Nov 10;120(46):e2308911120. doi: 10.1073/pnas.2308911120 (PMC10655579; doi:10.1073/pnas.2308911120)
Supplement: Supplementary file 1 — Appendix 01 (PDF) [file pnas.2308911120.sapp.pdf]

Supporting Information for

Emotions and courtship help bonded pairs cooperate, but emotional agents are vulnerable to deceit

Suzanne Sadedin, Edgar A. Duéñez-Guzmán and Joel Z. Leibo

E-mail: suzanne.sadedin@gmail.com

This PDF file includes:

Supporting text
Figs. S1 to S9
Tables S1 to S4

Supporting Information Text

Code and data associated with this submission will be made available in GitHub under a shared research repository. Large datafiles will be hosted on a Cloud bucket for download by the public with instructions available in the documentation of the GitHub repository.

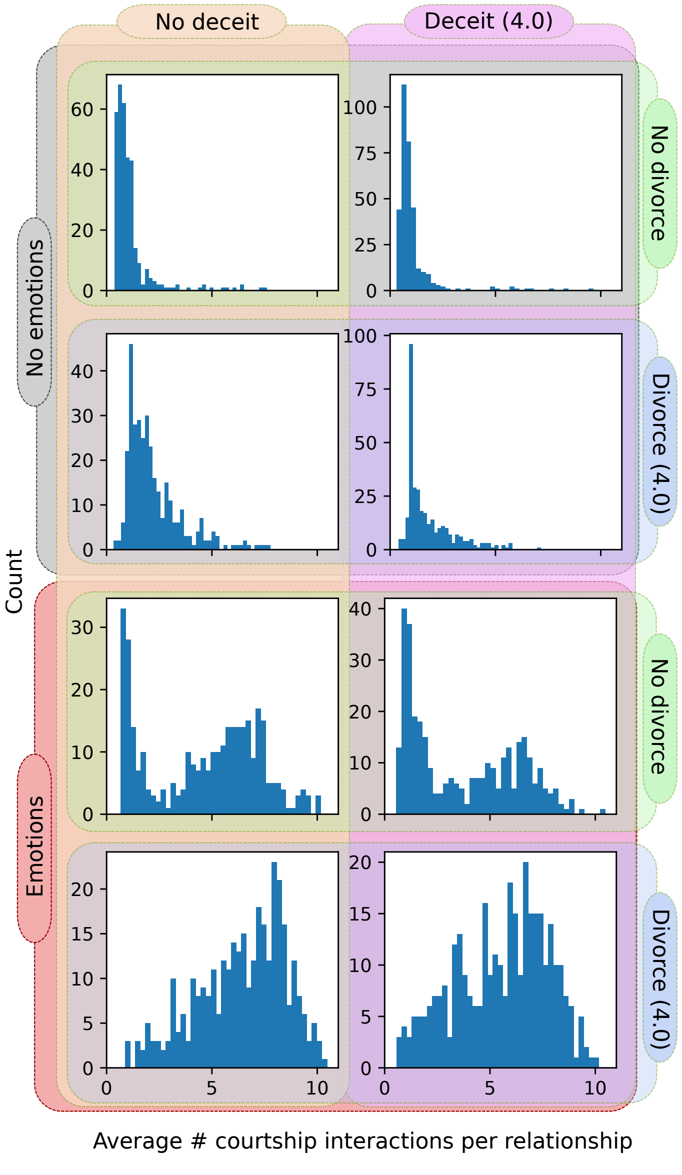

Fig. S1. Average number of courtship interactions in new relationships, across all noise levels. The panels show results with and without emotional bookkeeping, costly (4.0) divorce, and costly (1.6) deceit.

Table S1. Statistics of the average number of courtship interactions in new relationships.

Statistic	Value	Statistic	Value
No emotion, No divorce, No deceit		No emotion, No divorce, Deceit	
mean	1.114310	mean	1.072991
std	1.022322	std	1.124746
min	0.369759	min	0.298363
25%	0.609761	25%	0.606072
50%	0.862630	50%	0.782272
75%	1.139643	75%	1.043623
max	7.608198	max	9.641941
No emotion, Divorce, No deceit		No emotion, Divorce, Deceit	
mean	2.243758	mean	1.832162
std	1.321713	std	1.149524
min	0.322912	min	0.400312
25%	1.289583	25%	1.011979
50%	1.853309	50%	1.360567
75%	2.799770	75%	2.318183
max	7.778719	max	7.152656
Emotion, No divorce, No deceit		Emotion, No divorce, Deceit	
mean	4.616646	mean	3.698379
std	2.633642	std	2.511906
min	0.673672	min	0.553750
25%	1.630047	25%	1.252979
50%	5.178601	50%	3.108352
75%	6.709848	75%	5.960208
max	10.175371	max	10.484043
Emotion, Divorce, No deceit		Emotion, Divorce, Deceit	
mean	6.423701	mean	5.550036
std	2.089011	std	2.197705
min	0.885086	min	0.575172
25%	5.023483	25%	3.853826
50%	6.739450	50%	5.898719
75%	8.053592	75%	7.270398
max	10.465187	max	10.165112

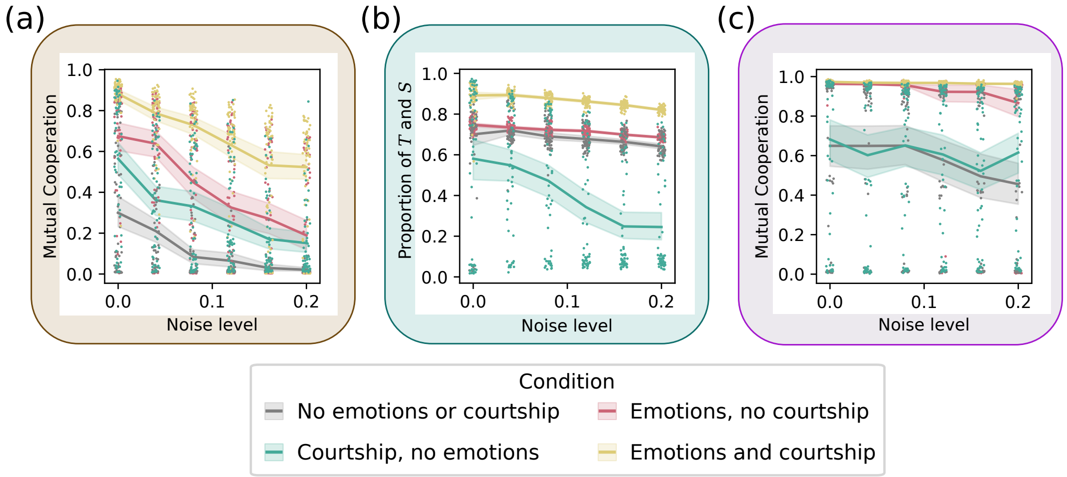

Fig. S2. Variations on the model for the baseline scenario (costly divorce, no deceit $m = 0.01, c_{\mathtt{div}} = 4.0, c_{\mathtt{cou}} = 0.0$). (a) Using a smooth sigmoid curve for decisions instead of a piecewise linear one does not influence results. (b) Results with Snowdrift payoffs ($R = 1, T = 3, S = 1, P = -1$) where T and S maximise social welfare. In this case we do not see high levels of R, but instead T and S are often very high. Across conditions, T and S are highest when both emotions and courtship are allowed. Emotions without courtship are slightly beneficial to T/S, but courtship without emotions reduces it. (c) Under Staghunt payoffs ($R = 3, T = 1, S = -1, P = 1$), courtship alone has little benefit to cooperation, but emotional bookkeeping does, and their combination yields even higher cooperation.

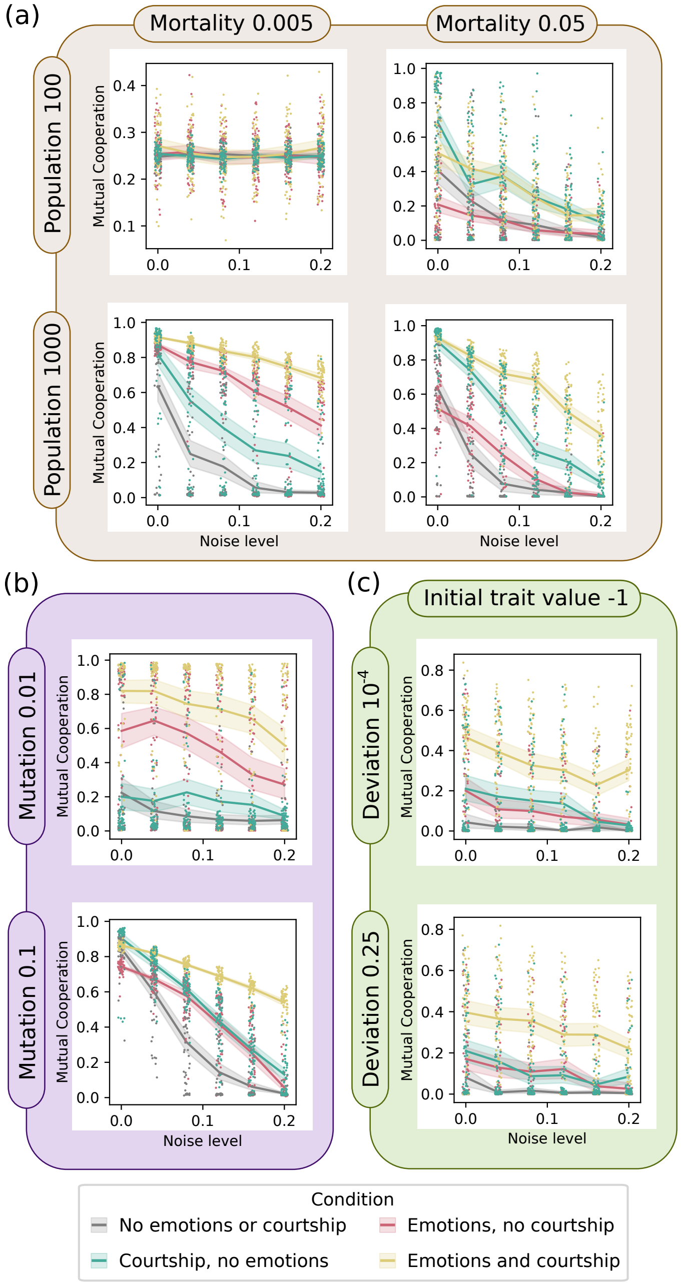

Fig. S3. Sensitivity analysis. Combining emotions and courtship leads to increased cooperation across a wide range of scenarios. Results are for baseline scenario (costly divorce, no deceit $m = 0.01, c_{\mathtt{div}} = 4.0, c_{\mathtt{cou}} = 0.0$), with variants as follows. (a) Population ($Pop = 1000, 100$) and mortality ($m = 0.005, 0.05$). Evolution of cooperation is more successful in larger populations with lower mortality, and does not occur in small populations with very low mortality. At high mortality, courtship is more effective and emotions less. This is unsurprising since the benefits of cooperation and emotional bookkeeping accrue over long relationships. (b) Mutation rate $\sigma = 0.01, 0.1$; extremely high mutation rates increase cooperation in the absence of courtship (c) All initial trait values = -1.0, initial variance = 0.0001, 0.25. Cooperation takes longer to emerge when the initial population is strongly biased against cooperation and relationships, but still benefits from emotions and courtship.

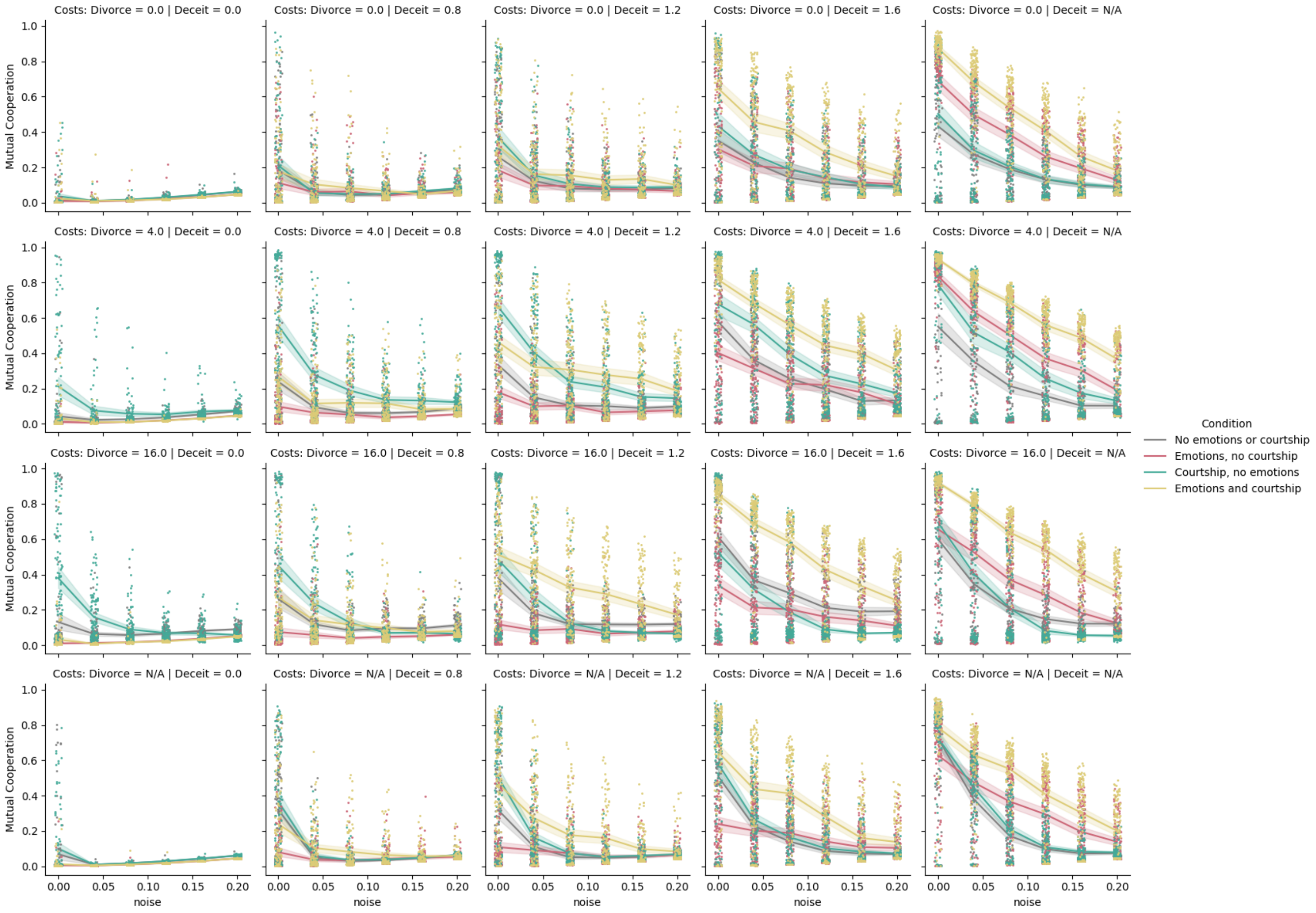

Fig. S4. Action noise scenario: mutual cooperation rate with and without emotions and courtship graphed against noise level. Panels indicate increasing deceit cost (left to right) and increasing divorce cost (top to bottom). N/A indicates that divorce or deceit was disallowed (bottom row / rightmost column respectively).

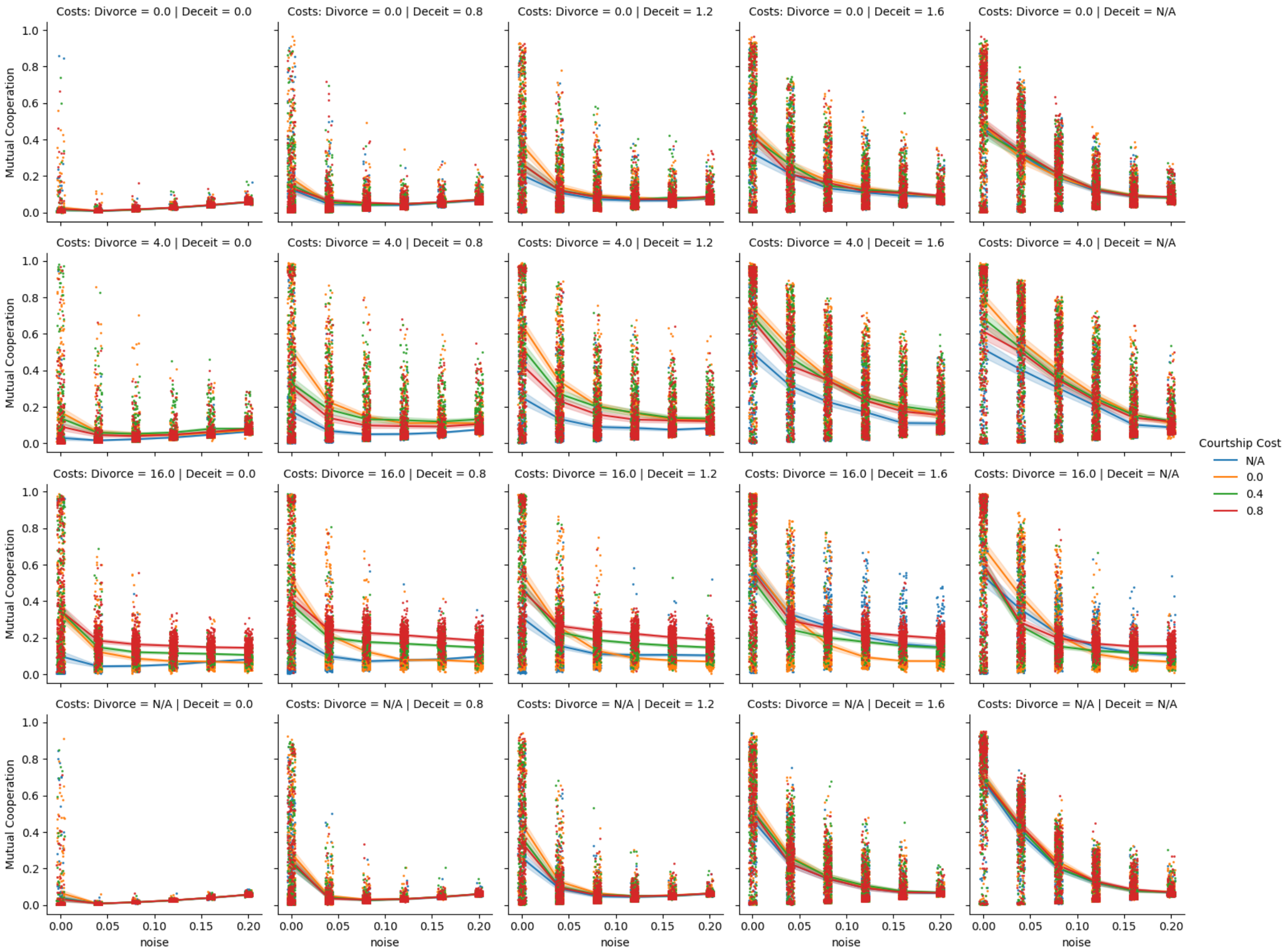

Fig. S5. Mutual cooperation rate with different courtship costs graphed against noise level without emotions for action noise. Panels indicate increasing deceit cost (left to right) and increasing divorce cost (top to bottom).

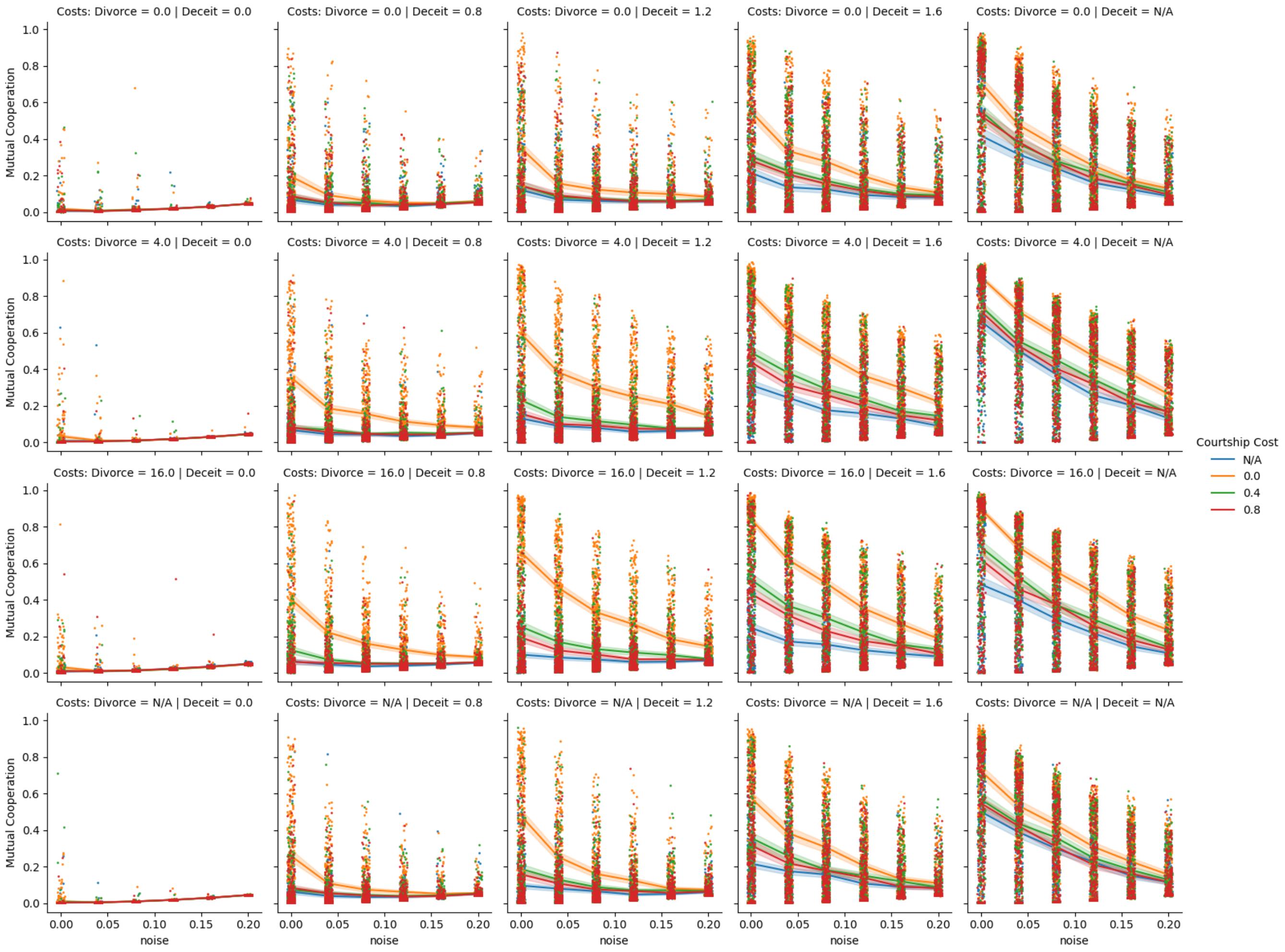

Fig. S6. Mutual cooperation rate with different courtship costs graphed against noise level with emotions for action noise. Panels indicate increasing deceit cost (left to right) and increasing divorce cost (top to bottom).

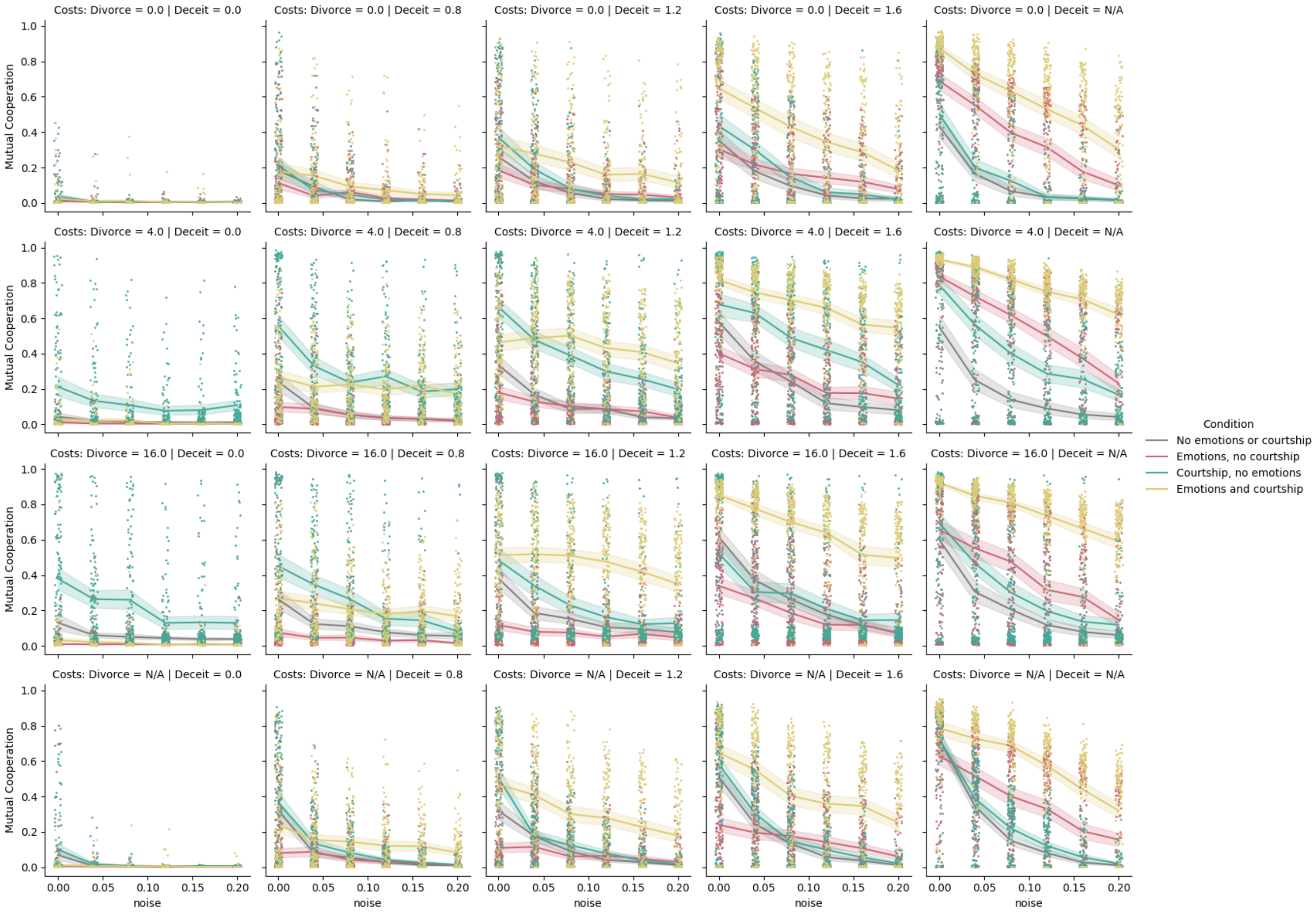

Fig. S7. Perceptual noise scenario: Mutual cooperation rate with and without emotions and courtship graphed against noise level. Panels indicate increasing deceit cost (left to right) and increasing divorce cost (top to bottom). N/A indicates that divorce or deceit was disallowed (bottom row / rightmost column respectively).

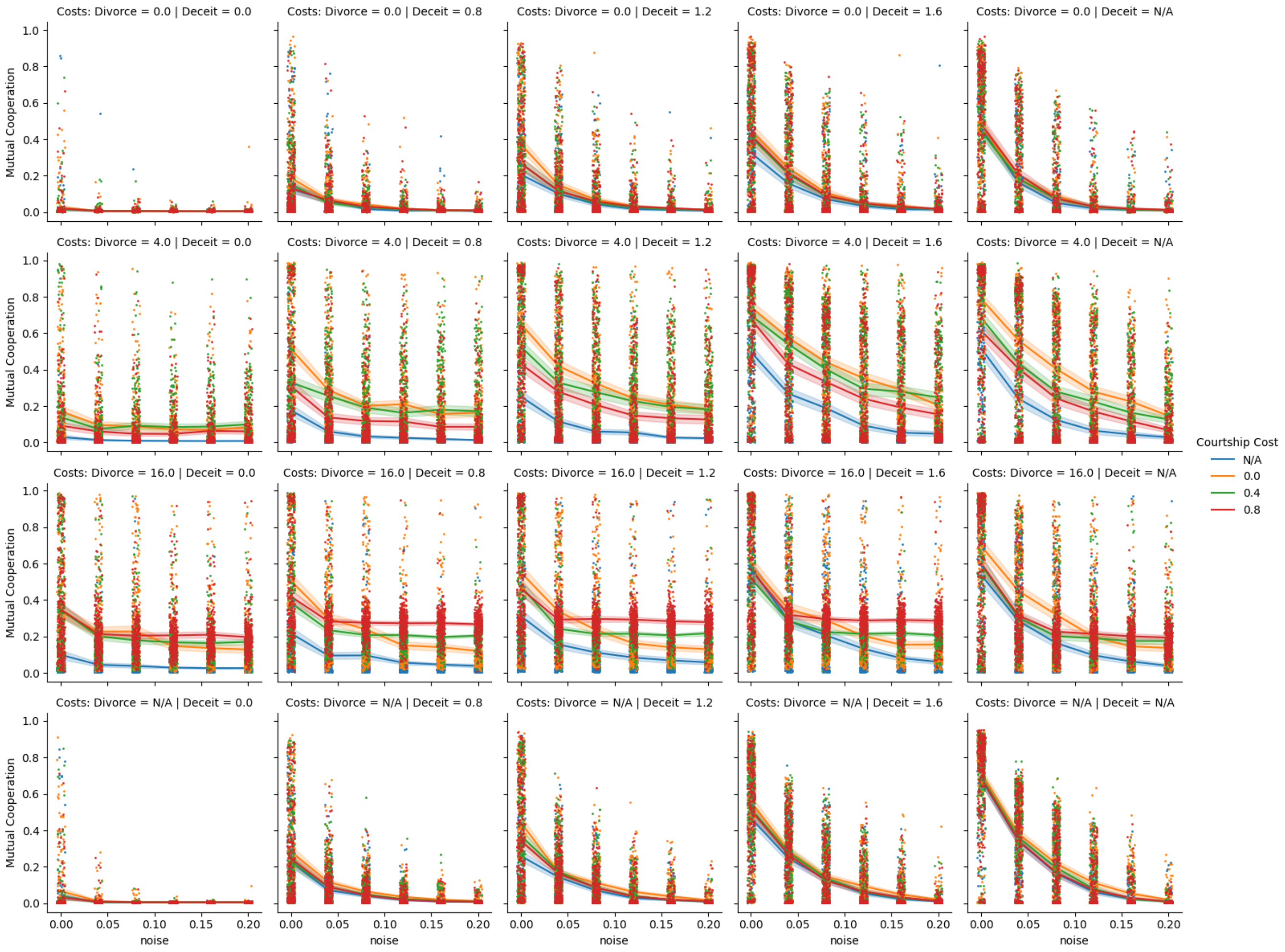

Fig. S8. Mutual cooperation rate with different courtship costs graphed against noise level without emotions for perceptual noise. Panels indicate increasing deceit cost (left to right) and increasing divorce cost (top to bottom). N/A indicates that divorce or deceit was disallowed (bottom row / rightmost column respectively).

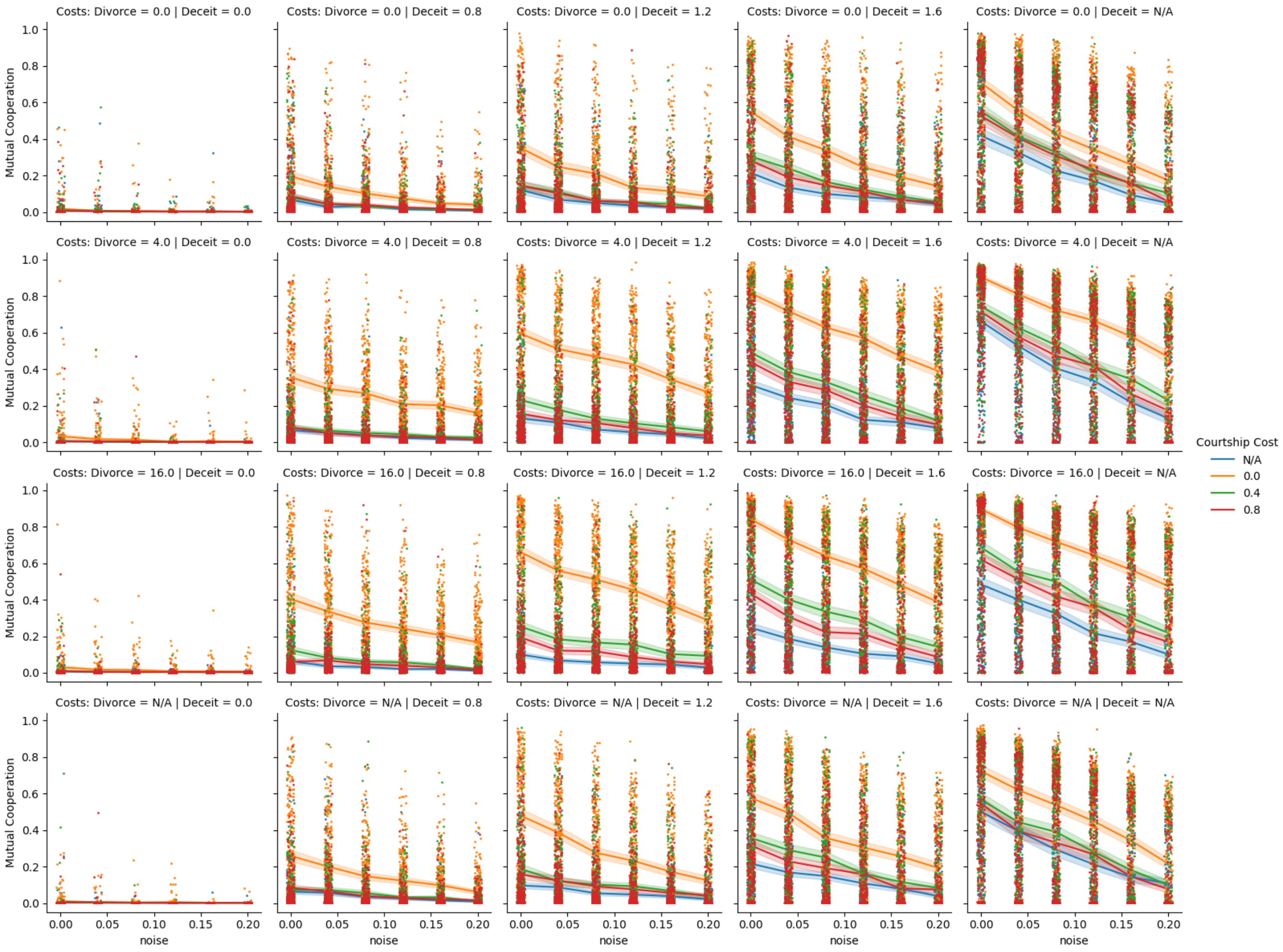

Fig. S9. Mutual cooperation rate with different courtship costs graphed against noise level with emotions for perceptual noise. Panels indicate increasing deceit cost (left to right) and increasing divorce cost (top to bottom). N/A indicates that divorce or deceit was disallowed (bottom row / rightmost column respectively).

Table S2. Multivariate linear model for effect of factors deceit, courtship, emotions, and noise level on strategy frequency

	Value	Num DF	Den DF	F value	Pr $> F$
Intercept					
Wilks' lambda	-0.000	12.000	1,136.000	-4,584,309,298,111,998.000	1.000
Pillai's trace	1.000	12.000	1,136.000	-4,584,309,298,111,998.000	1.000
Hotelling-Lawley trace	-48,425,802,444,845.047	12.000	1,136.000	-4,584,309,298,111,998.000	1.000
Roy's greatest root	-48,425,802,444,845.047	12.000	1,136.000	-4,584,309,298,111,998.000	1.000
allowdeceit					
Wilks' lambda	0.970	11.000	1,137.000	3.242	0.000
Pillai's trace	0.030	11.000	1,137.000	3.242	0.000
Hotelling-Lawley trace	0.031	11.000	1,137.000	3.242	0.000
Roy's greatest root	0.031	11.000	1,137.000	3.237	0.000
allowplay					
Wilks' lambda	0.881	11.000	1,137.000	13.975	0.000
Pillai's trace	0.119	11.000	1,137.000	13.975	0.000
Hotelling-Lawley trace	0.135	11.000	1,137.000	13.975	0.000
Roy's greatest root	0.135	11.000	1,137.000	13.975	0.000
emotions					
Wilks' lambda	0.808	11.000	1,137.000	24.607	0.000
Pillai's trace	0.192	11.000	1,137.000	24.607	0.000
Hotelling-Lawley trace	0.238	11.000	1,137.000	24.607	0.000
Roy's greatest root	0.238	11.000	1,137.000	24.607	0.000
noise					
Wilks' lambda	0.862	11.000	1,137.000	16.600	0.000
Pillai's trace	0.138	11.000	1,137.000	16.612	0.000
Hotelling-Lawley trace	0.161	11.000	1,137.000	16.591	0.000
Roy's greatest root	0.160	11.000	1,137.000	16.530	0.000

Table S3. Post-hoc tests: effect of factors deceit, courtship, emotions, and noise level on relative frequency of generous strategies using Šidák-corrected p-values

Cooperator				
	Sum of squares	df	F value	Pr $> F$
Deceit	0.000	1.000	0.027	1.000
Courtship	0.131	1.000	17.234	0.000
emotions	0.000	1.000	0.047	1.000
PriorEmotion	0.715	3.000	31.386	0.000
Noise	0.120	1.000	15.868	0.001
Residual	1.609	212.000	nan	nan
**Generous Opportunist**				
	Sum of squares	df	F value	Pr $> F$
Deceit	0.003	1.000	1.306	0.828
Courtship	0.000	1.000	0.029	1.000
emotions	0.024	1.000	9.367	0.015
PriorEmotion	0.046	3.000	6.027	0.004
Noise	0.001	1.000	0.399	0.989
Residual	0.539	212.000	nan	nan
**Generous TFT**				
	Sum of squares	df	F value	Pr $> F$
Deceit	0.000	1.000	0.021	1.000
Courtship	0.002	1.000	0.490	0.981
emotions	0.054	1.000	17.180	0.000
PriorEmotion	0.191	3.000	20.243	0.000
Noise	0.000	1.000	0.007	1.000
Residual	0.668	212.000	nan	nan
**Generous Trigger**				
	Sum of squares	df	F value	Pr $> F$
Deceit	0.001	1.000	0.362	0.991
Courtship	0.005	1.000	1.846	0.686
emotions	0.001	1.000	0.449	0.985
PriorEmotion	0.053	3.000	6.012	0.004
Noise	0.015	1.000	5.089	0.141
Residual	0.621	212.000	nan	nan
**Generous WSLS-like**				
	Sum of squares	df	F value	Pr $> F$
Deceit	0.007	1.000	3.440	0.332
Courtship	0.002	1.000	0.925	0.915
emotions	0.000	1.000	0.035	1.000
PriorEmotion	0.043	3.000	7.334	0.001
Noise	0.000	1.000	0.000	1.000
Residual	0.417	212.000	nan	nan
**Quitter**				
	Sum of squares	df	F value	Pr $> F$
Deceit	0.002	1.000	0.442	0.986
Courtship	0.012	1.000	2.528	0.514
emotions	0.010	1.000	1.993	0.648
PriorEmotion	0.117	3.000	7.982	0.000
Noise	0.004	1.000	0.882	0.924
Residual	1.037	212.000	nan	nan

Table S4. Post-hoc tests: effect of factors deceit, courtship, emotions, and noise level on relative frequency of non-generous strategies using Šidák-corrected p-values

Defector				
	Sum of squares	df	F value	Pr $> F$
emotions	0.161	1.000	5.120	0.139
Deceit	0.298	1.000	9.475	0.014
Courtship	0.635	1.000	20.175	0.000
PriorEmotion	1.130	3.000	11.968	0.000
Noise	0.677	1.000	21.494	0.000
Residual	6.675	212.000	nan	nan
NoS				
	Sum of squares	df	F value	Pr $> F$
emotions	0.000	1.000	0.002	1.000
Deceit	0.014	1.000	0.504	0.980
Courtship	0.199	1.000	7.378	0.042
PriorEmotion	0.401	3.000	4.955	0.014
Noise	0.207	1.000	7.686	0.036
Residual	5.713	212.000	nan	nan
Opportunist				
	Sum of squares	df	F value	Pr $> F$
emotions	0.016	1.000	0.751	0.947
Deceit	0.079	1.000	3.673	0.295
Courtship	0.000	1.000	0.000	1.000
PriorEmotion	0.208	3.000	3.238	0.131
Noise	0.029	1.000	1.335	0.821
Residual	4.537	212.000	nan	nan
Reciprocator				
	Sum of squares	df	F value	Pr $> F$
emotions	0.054	1.000	2.583	0.501
Deceit	0.007	1.000	0.357	0.992
Courtship	0.124	1.000	5.975	0.089
PriorEmotion	0.324	3.000	5.202	0.010
Noise	0.105	1.000	5.052	0.144
Residual	4.403	212.000	nan	nan
Trigger				
	Sum of squares	df	F value	Pr $> F$
emotions	0.056	1.000	2.023	0.640
Deceit	0.006	1.000	0.204	0.998
Courtship	0.031	1.000	1.130	0.871
PriorEmotion	1.456	3.000	17.600	0.000
Noise	0.048	1.000	1.747	0.713
Residual	5.846	212.000	nan	nan
WSLS				
	Sum of squares	df	F value	Pr $> F$
emotions	0.000	1.000	0.007	1.000
Deceit	0.034	1.000	3.083	0.396
Courtship	0.000	1.000	0.013	1.000
PriorEmotion	0.055	3.000	1.644	0.697
Noise	0.090	1.000	8.174	0.028
Residual	2.344	212.000	nan	nan
